# Towards Phosphate Detection in Hydroponics Using Molecularly Imprinted Polymer Sensors

**DOI:** 10.3390/s18020531

**Published:** 2018-02-10

**Authors:** Christopher S. Storer, Zachary Coldrick, Daniel J. Tate, Jack Marsden Donoghue, Bruce Grieve

**Affiliations:** 1 School of Electrical & Electronic Engineering, University of Manchester, Oxford Road, Manchester M13 9PL, UK; christopher.storer@outlook.com (C.S.S.); zachary.coldrick@manchester.ac.uk (Z.C.); 2 Organic Materials Innovation Centre, School of Chemistry, University of Manchester, Manchester, M13 9PL, UK; daniel.tate@manchester.ac.uk; 3 School of Materials, University of Manchester, Oxford Road, Manchester M13 9PL, UK; jack.donoghue@manchester.ac.uk

**Keywords:** hydroponics, interdigitated electrodes, molecularly imprinted polymer, nutrient monitoring, phosphate, polymer sensor, precision agriculture

## Abstract

An interdigitated electrode sensor was designed and microfabricated for measuring the changes in the capacitance of three phosphate selective molecularly imprinted polymer (MIP) formulations, in order to provide hydroponics users with a portable nutrient sensing tool. The MIPs investigated were synthesised using different combinations of the functional monomers methacrylic acid (MAA) and *N*-allylthiourea, against the template molecules diphenyl phosphate, triethyl phosphate, and trimethyl phosphate. A cross-interference study between phosphate, nitrate, and sulfate was carried out for the MIP materials using an inductance, capacitance, and resistance (LCR) meter. Capacitance measurements were taken by applying an alternating current (AC) with a potential difference of 1 V root mean square (RMS) at a frequency of 1 kHz. The cross-interference study demonstrated a strong binding preference to phosphate over the other nutrient salts tested for each formulation. The size of template molecule and length of the functional monomer side groups also determined that a short chain functional monomer in combination with a template containing large R-groups produced the optimal binding site conditions when synthesising a phosphate selective MIP.

## 1. Introduction

Within the field of precision agriculture, the accurate in-field measurement of the macronutrients nitrate, phosphate, and potassium in soils and hydroponic growth media is a vital component to controlling crop yields and plant disease levels [1,2]. The role of phosphate is particularly important in cellular metabolism and the production of nucleic acids, with a phosphorous (P) deficiency resulting in the stunted growth of plants and poor development of root systems [3]. However, an overdosing of the nutrient through phosphate rich fertilisers leads to significant leaching of labile phosphate into the local water table. This leads to a population explosion in species of blue algae, and subsequently the eutrophication of nearby ponds and water supplies [4]. As such, the control of phosphate content in the environment is crucial.

Yet, a significant challenge within the research and development community has been the determination of a portable sensor design that can selectively measure the concentration of inorganic phosphate present within a growth media. Phosphate displays cross-interference with other common nutrient cations such as NO_3_^−^ when measured using conventional electrochemical sensors based on electro-conductivity (EC) measurements or ion selective electrodes (ISEs) [5]. This is due to the structure of the orthophosphate ion, with the central P atom covalently bonded to four oxygen atoms, creating a hydrophilic sphere around the anion and resulting in a high enthalpy of hydration, making it difficult to detect. Another challenge to detection is that the structure of the phosphate molecule present is highly dependent on the pH of the environment, where it exists as H_2_PO_4_^−^ and H_3_PO_4_ in acidic environments, whereas it takes the forms of PO_4_^3−^ and HPO_4_^2−^ in alkaline environments [5]. Both of these factors combine to result in the phosphate occupying a low position on the Hofmeister Selectivity Series for anions in salts. This produces a selectivity order of ClO_4_^−^ > I^−^ > NO_3_^−^ > Br > Cl^−^ > F^−^ > H_2_PO_4_^−^ > SO_4_^2−^ in electrode measurements of nutrient growth media [6,7,8].

A solution to the challenges associated with cross-interference is the introduction of a molecularly imprinted polymer (MIP) as a selective sensor recognition element. MIPs are biomimetic materials that contain three-dimensional binding sites similar to those of enzymes used in biosensors [9]. An MIP consists of three key components: a crosslinked hydrocarbon polymer network that provides structure to the material; a functional monomer containing side groups that allow for non-covalent bonding interactions at the binding sites; and a template molecule around which the binding site forms [10].

**Figure 1:**
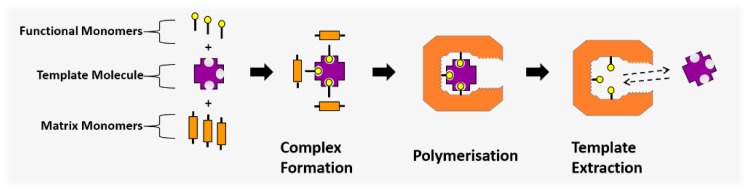
The molecular imprinting process, where functional monomer ligands form a coordination complex around the template molecule and are polymerised in place to create a complimentary binding site.

The MIP structure is synthesised by means of a molecular imprinting process, where the binding site is created. The template molecule acts as a coordination centre in a complex formation, with the functional monomers acting as ligands since they contain functional groups that allow for dipole interactions and hydrogen bonding with the template molecule. The ligand structure is then fixed in place by polymerising the functional monomers with a second, crosslinking monomer component. This completes the imprinting process (Figure 1), creating a complimentary binding site to the template molecule.

Kugimiya et al. successfully demonstrated the ability to imprint phosphate in a series of papers where an MIP was created using a diphenyl phosphate template with a binding site based on *N*-allylthiourea for applications in water remediation and phosphate recovery [11,12,13,14]. More recently, Quint et al. further established the suitability of phosphate selective MIPs for use as sensing elements by using carbon nanotubes as a method for electrical transduction to detect the binding event of a phosphate MIP using dipentyl phosphate as the template molecule and methacrylic acid (MAA) as the functional monomer [15]. While Qunit et al. employed voltammetry to determine the concentration of phosphate, this Faradaic technique can be interfered with by dissolved oxygen, electrochemical side reactions, and electrode fouling. The study presented here attempts to use changes in dielectric properties of the MIP interrogated in a non-Faradaic and non-destructive fashion when the MIP thin film covers the interdigitated transducer to determine the presence of bound phosphate.

**Figure 2:**
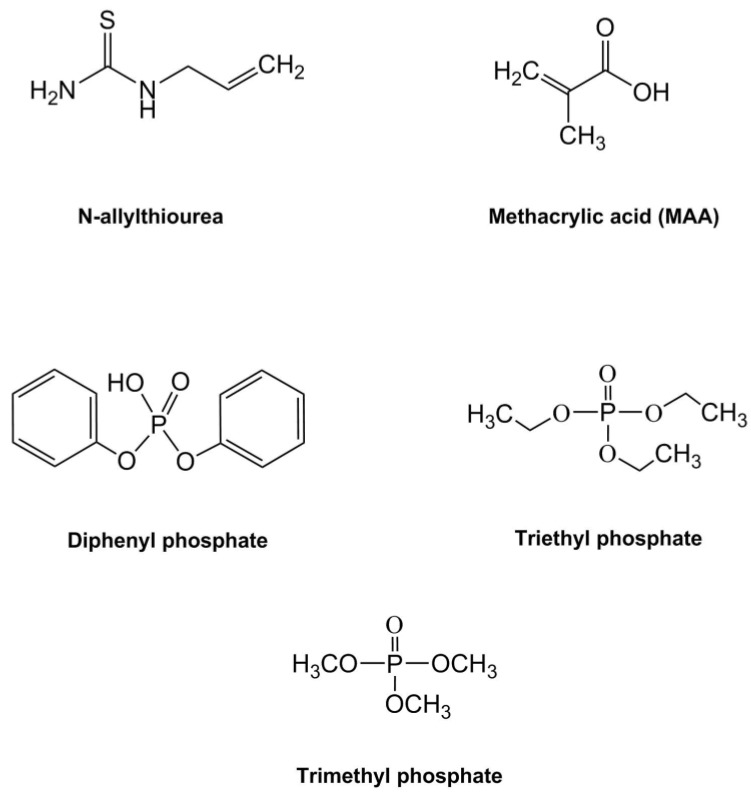
Chemical structures for the functional monomers (methacrylic acid-MAA, and *N*-allylthiourea) and the template molecules (diphenyl phosphate, triethyl phosphate, and trimethyl phosphate) investigated in this study.

For this investigation, three MIP formulations were synthesised from combinations of *N*-allylthiourea and MAA as the functional monomers, with diphenyl phosphate, triethyl phosphate, and trimethyl phosphate selected for testing as the template molecules (Figure 2). The three template molecules were chosen to provide a range of low molecular weight, three-dimensional structures with a central phosphate ion to create complementary binding sites of varying affinity to the phosphate ion.

The transduction of the MIPs was carried out using a non-destructive electrical technique, using an array of interdigitated electrodes to measure the change in capacitance of the MIP sensing materials [16]. This provides a measurement of the MIP binding to the analyte by observing a change in the dielectric constant of the material as binding sites become occupied within the MIP structure, resulting in the capacitance shift. The system was designed with the ultimate aim of integration of the MIP sensor within an in-line unit that could be connected to a recirculating hydroponics setup. This would allow for the real-time monitoring and dosing of individual macronutrients in response to the variation in plant nutrition requirements during their individual growth and development stages prior to harvest.

## 2. Materials and Methods

The aim of this investigation was to produce an interdigitated electrode array for the application of measuring a phosphate responsive MIP-based sensor.

### 2.1. Reagents and Apparatus

The following reagents were used for this study and were purchased from Sigma-Aldrich (Gillingham, UK) including the solvents: acetone, propan-2-ol, methanol, toluene, and diglycol methyl ether (diglyme), all of analytical grade. 3-(trimethoxysilyl)propyl methacrylate, 98%, was purchased as a silanisation source. For the MIP formulations, poly(methyl methacrylate) (PMMA), with a molecular weight of 996,000, and trimethylolpropane trimethacrylate (TRIM) were used for the network and crosslinking monomers. Bis[4-(dimethylamino)phenyl]methanone (known by the trade name Michler’s Ketone) was selected as the photoinitiator for polymerisation. For the MIP functional monomers, *N*-allylthiourea 98% and MAA 99% were selected, whilst diphenyl phosphate, triethyl phosphate, and trimethyl phosphate were selected for the template molecule. For the nutrient salts, sodium dihydrogen phosphate (NaH_2_PO_4_), sodium nitrate (NaNO_3_), and sodium sulfate (Na_2_SO_4_). The deionised water used for producing the aqueous phosphate, nitrate, and sulfate salt solutions was produced in-lab using a Millipore Direct-Q 3 Smart water purification system.

Polymerisation was carried out using an OmniCure LX400+ (Excelitas Technologies, Waltham, MA, USA) LED spot curing system to deliver 9.5 Wcm-2 source UV radiation at a wavelength of 365nm via an optical fibre cable. Spin-coating of substrates with the polymer formulations used a Laurell Technologies WS-400 series spin coater (Laurell Technologies, North Wales, PA, USA) with a micro controller.

Inductance, capacitance, and resistance (LCR) testing was carried out using a Hewlett Packard 4284A Precision LCR Meter (20 Hz–1.0 MHz) (Keysight Technologies UK, Wokingham, UK). The chromium-quartz photomask used in the electrode production was built to order based on a design specification sent to third-party manufacturer Compugraphics International Ltd (Fife, UK).

### 2.2. Photopolymerisation and Spin-Coating of MIP/NIP Materials

A spin-coating method was devised to allow for several MIP formulations to be created, allowing for interchangeable functional monomers and template molecule components. This was adapted from a procedure published by Schmidt, Mosbach and Haupt [17] that allowed for the photopolymerisation of smooth, thin, and porous MIP films using a porogen agent. Diglyme was selected as both the suspension solvent for the monomer mixture, and as the porogen. PMMA and TRIM were selected for the polymer’s network and crosslinking monomer components, with Michler’s Ketone selected as the photoinitiator for the UV-induced free radical polymerisation reaction. These were used to produce three stock solutions prior to spin-coating, which would allow for the functional monomers and templates in be interchanged. These included: a network polymer solution (NPS), an initiator solution (IS), and a functional monomer and template solution (FMTS) (Table 1). 

A final spin-coating mixture was then prepared from the three stock solutions (Table 2), producing a 2 mL solution that contained 0.311 mol dm^-3^ of the functional monomer and 0.0313 mol dm^-3^ of the template molecule. This is a result of a dilution factor of 0.1875 being applied to the FMTS as it is added to the spin-coating mixture.

The spin-coating solution was passed through a polytetrafluoroethylene (PTFE) filter with a pore size of 45 µm to provide a particulate-free solution. This method was followed for each of the three MIP formulations produced with varying binding site structures, and were labelled MIP1, MIP2, and MIP3 (Table 3) to differentiate between the functional monomer and template combinations used.

**Figure 3:**
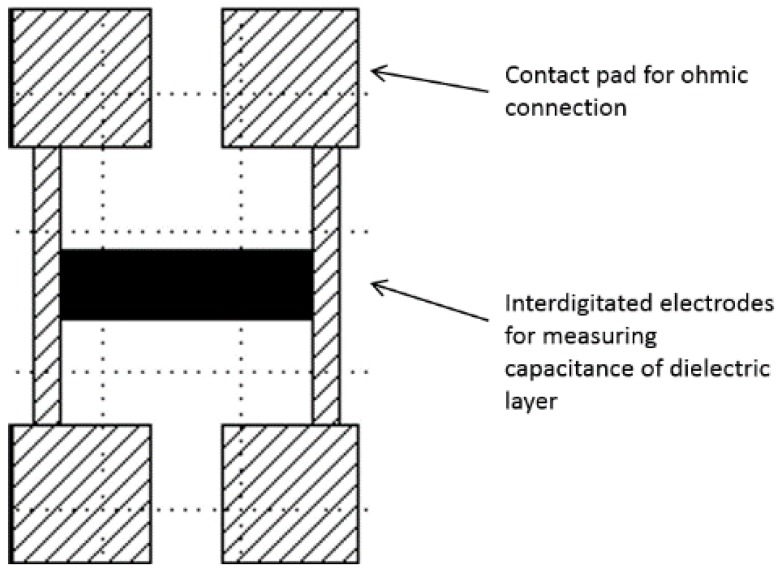
Schematic of the chromium-quartz interdigitated electrode device, with a central electrode array connected to four ohmic contact pads to create an interface with a Hewlett Packard 4284A Precision LCR meter.

Spin-coating of the MIP formulations took place within a nitrogen glovebox using a Laurell Technologies WS-400 series spin-coater. The substrate electrode device was secured in place using a vacuum chuck within the spin-coater, and 200 µL of the filtered spin-coating mixture was dispensed onto the electrodes in the centre of the substrate (Figure 3). The substrate was then spun for 180 s at a speed of 3000 rpm, with an OmniCure UV LED MAX Head (Excelitas Technologies, Waltham, MA, USA) wired into the lid of the spin-coater and simultaneously triggered to emit UV radiation at a wavelength of 365 nm and intensity of 9 W cm^−2^ for 180 s.

Upon completion of the curing cycle, the LED and vacuum chuck were deactivated and the polymer-coated substrate was removed from the spin-coater for storage and subsequent testing. A control polymer was also produced, known as a non-imprinted polymer (NIP), consisting purely of the polymer network components (PMMA and TRIM) in diglyme, and it was polymerised using the same photoinitiator. This allowed for a baseline to be produced to act as a control material that all imprinted materials were compared to throughout the testing process. The above process coated the entire quartz device with a thin layer of the appropriate NIP/MIP polymer. The device was then held in a specially designed cell with a rubber O-ring around the interdigitated electrodes to protect it from acetone vapor, while the connection pads were wiped clean of polymer using a paper towel soaked in acetone to allow for electrical connection to the contact pads.

### 2.3. Microfabrication of Interdigitated Electrode Substrate

The electrode devices for measuring the capacitance of the MIP layer were constructed with quartz glass as the insulating substrate and chromium for the conducting electrode tracks. The devices were designed in-house at the University of Manchester and then microfabricated externally by third party contractor Compugraphics International Limited. They were produced using the same photoresist and etching fabrication process as a chromium-quartz photomask used in photolithography. This produced a 6 inch by 6 inch by 0.12 inch photomask with a chrome layer thickness of 100 nm, containing 15 identical electrode designs. The photomask was then cut into 15 separate devices by another third-party contractor, Loadpoint, using a diamond-tipped saw.

**Figure 4:**
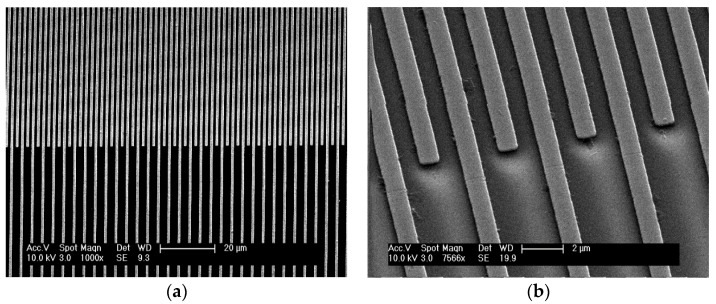
Secondary electron SEM images of the interdigitated electrode array at (**a**) low, and (**b**) high, magnification. The images show the device to be free of defects and manufactured to a feature size of 1 µm.

The interdigitated electrode design consisted of 500 electrode lines of 100-nm thick chromium, 1 cm long and 1 µm wide, with a separation of 1 µm between individual electrode digits. The two electrode combs were separated and connected to a pair of contact pads (Figure 4) that allowed for interfacing the connectors with the Hewlett Packard 4284A Precision LCR meter. Contact was made to the contact pads using metal mesh Shielding Strip of Nickel Copper Alloy tape with a polyurethane core, 1 m × 8 mm × 5 mm, (DRE8x5NI-N4V0-1000, RS components, Corby, UK) cut to a size to completely cover the pads with crocodile clips applying pressure and interfacing via common earth Bayonet Neill-Concelman (BNC) cables.

**Figure 5:**
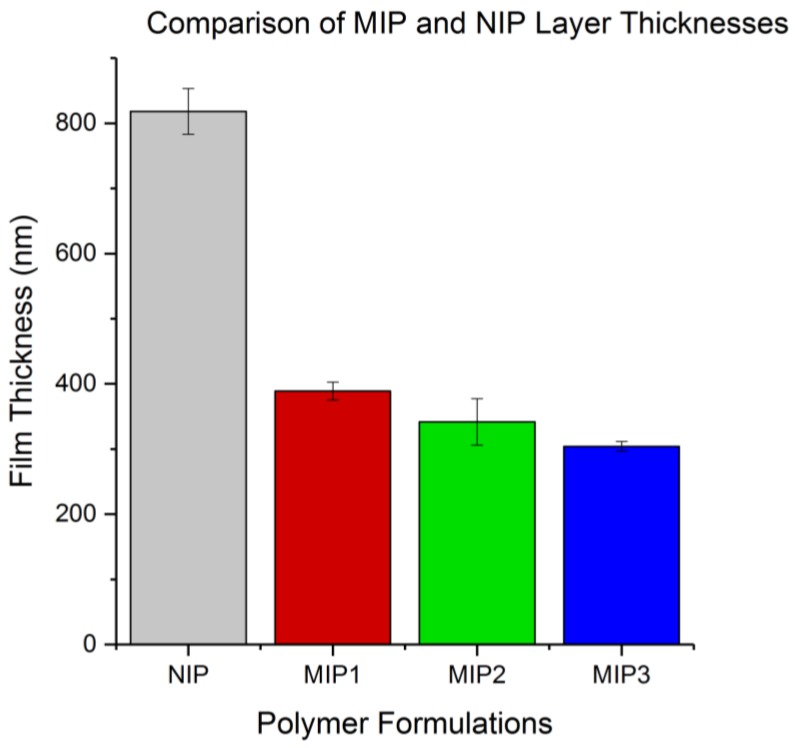
A comparison of the spin-coated non-imprinted polymer (NIP) and MIP layer thicknesses produced using a Bruker profilometer.

Following the microfabrication of the devices, scanning electron microscopy (SEM) was used to inspect the devices to ensure that the electrodes tracks were free of defects and inter-electrode bridging, as well as to ensure that the tracks were uniform (Figure 5). Prior to spin-coating, the chromium-quartz substrates then underwent a cleaning process to remove any dust or organic contaminants from their surface. Each device was cleaned using ultrasound whilst submerged sequentially in solutions of acetone, propan-2-ol, and methanol for 30 min per solvent, and then dried off using a nitrogen gun in a clean environment. 

The patterned chromium-quartz substrates were then silanised by immersion in a solution of 3-(trimethoxysilyl)propyl methacrylate and toluene for 24 h, to allow for bonding between the spin-coated polymer and the substrate during the photo-polymerisation of the MIP film.

### 2.4. Template/Analyte Extraction

Extraction of the template molecule from the MIP binding site was carried out via a diffusion-based method using multiple solvent washes. Methanol was used to remove the organophosphate templates, with each MIP-coated substrate submerged in 25 mL of methanol, with the solvent media being removed and changed three times over a 72-h period (i.e., with solvent changes every 24 h). This ensured that the template would be removed via a diffusion concentration gradient.

For the extraction of the nutrient salt analytes (phosphate, nitrate, or sulfate) during the cross-interference study, D.I. water was used to extract the analyte, following the same procedure described previously for methanol. This change in solvent was due to the ionic analytes being insoluble in an organic solvent. 

### 2.5. Capacitance Measurements

Capacitance measurements of the MIP-coated, planar interdigitated electrode array was carried out using a Hewlett Packard 4284A Precision LCR meter. Measurements were taken with an Alternating Current (AC) at a potential of 1.0 V root mean square (RMS) and a frequency of 1.0 kHz. An average of 100 recorded measurements were taken per sample and repeated in triplicate to provide error analysis. The LCR meter was connected to the electrode device contact pads using clips that were attached to four coaxial cables interfacing into the meter to provide analysis using the high potential, low potential, high current, and low current ports on the LCR terminal.

### 2.6. Thickness Measurements of Spin-Coated Polymer

Surface profile measurements for the polymer samples were carried out using a Bruker Dektak-XT profilometer (Bruker UK Limited, Coventry, UK) to determine the thickness of the MIP and NIP films. Separate substrates were prepared for surface profile measurement using the same 0.12-inch thick quartz substrate, but coated entirely on its top surface with a uniform, 100-nm thick layer of chromium and containing no electrode design. These were then spin-coated with the NIP, MIP1, MIP2, and MIP3 formulations following the same method as the electrode devices.

A ‘defect’ was introduced into the polymer films using a rounded stainless steel needle with a 1-mm diameter to mark a line transversely across the surface of the spin-coated substrate. This removes a thin strip of polymer, exposing the chromium beneath, but without marking the substrate itself. This allowed for the profilometer stylus to be dragged across the introduced gap and measure the change in height from the base (bare substrate) to the top of the film. Averages were taken over three samples per polymer formulation, with each sample measured in triplicate.

## 3. Results

### 3.1. Thickness of Polymer Film Results

By examining the thickness of the various polymer films produced from spin-coating, it was possible to observe the effect that altering the functional monomer and template pairing would have on the viscosity of the solution being spin-coated, and therefore the thickness of the film produced. The plotted results in Figure 5 show that the MIP formulations produced layers of similar thickness with a decreasing trend in MIP layer thickness moving from MIP1 to MIP3. The large disparity in thickness between the NIP and MIP layers is likely a result of higher viscosity of the NIP formulation compared to the NIPs, indicating that the MIP template molecules acted to decrease the viscosity for their respective formulation matrix prior to crosslinking. In the case of this study, the film thickness was measured to compare the effect of the different template molecules on the formulation viscosity and was not controlled. This parameter could be controlled in future experiments by the addition of the solvent diglyme to the more viscous NIP formulation to dilute the crosslinkable matrix. Additionally, increasing the speed and duration of the spin-coating process could produce layers of reduced thickness with the same viscosity. The NIP thickness was decided not to be of critical importance to the present study as a lack of nutrient salt binding was expected from the NIP. 

Each film formed a continuous layer over the chrome metal electrodes as intended, which was confirmed by observation under light microscopy. The polymer was observed to coat the electrodes and fill the 1-µm gaps, creating a series of micro capacitors similar to that of a parallel plate capacitor. This change in dielectric constant due to the polymer replacing air could be observed clearly using an LCR meter, and is reasonably consistent for each polymer raising the capacitance from 867 pF(±8 pF, 1 standard deviation, *n* = 1200) to 1150 pF (±30 pF, 1 standard deviation, *n* = 1200). This similarity between the films indicates that the major contribution of the polymer to the measured capacitance likely comes from the polymer situated directly between the electrode fingers with a lesser contribution from the polymer covering the top of the electrode and more distance from the interdigitated site. This is implied by the similarity of the capacitance of the NIP coating with the MIP coatings, despite the significant difference in film thickness.

### 3.2. Capacitance Changes in MIP/NIP Samples in Response to Phosphate, Nitrate, and Sulfate

The cross-interference study was carried out with the purpose of determining how specifically the three MIP formulations would bind to phosphate, and to what extent they would experience inference from other similarly sized nutrient anions. As such, the phosphate (PO_4_^−^) targeting MIPs were tested against the macronutrient nitrate (NO_3_^−^) and micronutrient sulfate (SO_4_^−^).

Capacitance measurements were carried out with an applied potential of 1 V RMS at 1 kHz after each of the following stages:

1. Bare Electrodes—untreated with no polymer or silanisation process applied.
2. Spin-Coated—measurements taken following spin-coating of the electrodes with an MIP or NIP formulation.
3. Methanol Wash—sample bathed in methanol to extract the template molecule from the polymer.
4. Phosphate—sample bathed in a 0.1 M solution of NaH_2_PO_4(aq)_ to provide exposure to the PO_4_^−^ anion.
5. D.I. Water Wash—sample bathed D.I water to extract PO_4_^−^ anion from the polymer.
6. Nitrate—sample bathed in a 0.1 M solution of NaNO_3(aq)_ to provide exposure to the NO_3_^−^ anion from the polymer.
7. D.I. Water wash—sample bathed in D.I. water to extract the NO_3_^−^ anion from the polymer.
8. Sulfate—sample bathed in a 0.1 M solution of Na_2_SO_4(aq)_ to provide exposure to the SO_4_^−^ anion.

**Figure 6:**
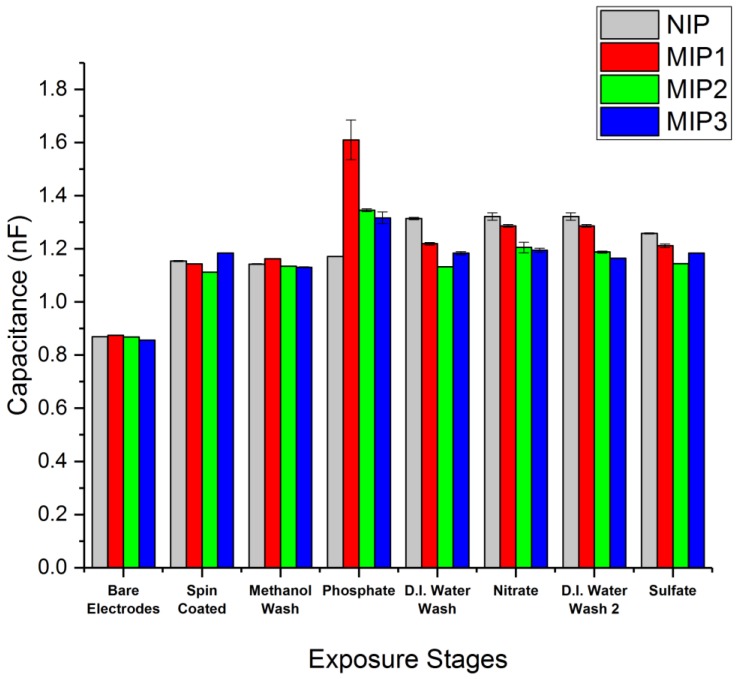
The MIP vs. NIP capacitance measurements at 1 V, 1 kHz for MIP1 (diphenyl phosphate and methacrylic acid), MIP2 (triethyl phosphate and methacrylic acid), and MIP3 (trimethyl phosphate and *N*-allylthiourea).

The devices were dried with a nitrogen gun immediately following removal from both the D.I. water and salt solutions in order to prevent a build-up of salt crystals. Each measurement was taken in triplicate across each device, with three devices used per MIP formulation, e.g., three devices coated in MIP, each measured three times, for a total of nine measurements per stage. Following this, the MIP capacitance measurements were plotted against the NIP results (Figure 6) to provide comparison, with standard deviation used to produce the error bars.

All three MIP formulations displayed a significant binding preference to phosphate over the nitrate and sulfate salts tested. MIP1 consisting of diphenyl phosphate paired with methacrylic acid displayed the largest shift in capacitance in the presence of phosphate (Figure 6). This was significantly above the capacitance observed for MIP2 (triethyl phosphate and methacrylic acid) and for MIP3 (trimethyl phosphate and *N*-allylthiourea). This increase may be due to a greater number of binding sites within MIP1 or a greater binding affinity in the MIP1 polymer to phosphate, thus allowing MIP1 to retain more phosphate after the methanol wash and subsequent drying compared to that of MIP2 and MIP3.

It was also observed that the NIP showed an increase in capacitance following exposure to the D.I. water wash. A possible reason for this could be an increase in the hydration of the NIP between the interdigitated electrodes, resulting in an increased dielectric constant of the material. Further investigation of this is required to draw a clear conclusion.

These preliminary results show that this technique is capable of measuring the binding of the phosphate to the MIP surfaces, and they also imply that the binding is reversible or that a relativity low affinity means the phosphate can be washed from the polymer. The affinity constant cannot be directly measured with the current data, but it is our intention to perform future concentration studies to determine the minimum concentration required to see a change in capacitance and determine the point at which each MIP becomes saturated. The observed reversibility upon washing also opens the door to an application within an aqueous flow cell where the capacitance of the MIP may give an indication as to the level of the phosphate ion at a particular time within a water flow.

A single device with bare electrodes (uncoated) was measured under the same conditions as above after drying under nitrogen and then with 200 µL of deionised water applied over the interdigitated electrode section of the device using a precision autopipette to form a drop completely covering the electrodes but not touching the contact pads. This process was repeated three times, with the electrode being dried under nitrogen gas flow between measurements.

The device capacitance measured 883 pF (±2 pF, 1 standard deviation, *n* = 150) while bare but increased to 22.5 mF (±11 nF, 1 standard deviation, *n* = 150) with the water drop covering the electrodes. This approximately 27-fold increase in capacitance was a result of the dielectric constant of water in comparison to air and is significantly higher than that of the electrodes with NIP/MIP coatings after exposure to water and salts. This measurement illustrates the large measured capacitance increase resulting from immersion in D.I. water and the reproducibility of the measurement without the MIP coverage.

## 4. Discussion and Conclusions

The three MIP formulations tested successfully demonstrated a specific binding affinity for phosphate quantified using the layer dielectric properties measured by capacitance, with the greatest capacitance increase observed for the MIP1 formulation based on a methacrylic acid functional monomer and diphenyl phosphate template combination. The other MIP formulations tested exhibited a smaller capacitance increase when exposed to phosphate, suggesting a lower affinity and thus a decreased ability to retain the phosphate ion subsequent to washing and drying. Each of the three MIP polymers showed a far smaller capacitance increase after exposure to solutions containing nitrate and sulphate ions, suggesting that a degree of specificity was achieved for each polymer with a preference for phosphate over other similarly sized and charged ions.

It was also observed that the thickness of the polymer film, whilst not having a large initial effect on the capacitance film measurements, did lead to a significant change in the mechanical properties of the polymer network, as seen by the increase in capacitance of the NIP following exposure to D.I. water. While the extra thickness of the NIP polymer compared with the MIPs might have resulted in greater impermeability to the salt solutions, this seems to be not that significant, as revealed by the increase in capacitance of the MIP subsequent to washing with D.I. water, indicating that it is at least permeable to water.

This initial study provides groundwork upon which it is the intention to carry out a further kinetic binding study to evaluate the affinity and number of assessable binding sites for the MIP1 polymer, along with an optimisation of the transduction interdigitated electrode device.

Further work to refine this sensor for full integration into a hydroponics system would be valuable, particularly targeting the further optimisation of the spin-coating process through a rheology study of the pre-spun monomer solutions to produce films of a uniform thickness across formulations. 

## Figures and Tables

**Table 1:** Molecularly imprinted polymer (MIP) formulation pre-spin stock solutions.

Stock Solution	Abbreviation	Content
Network Polymer Solution	NPS	1.6 mL of 10% wt PMMA in diglyme, and 1.115 mL TRIM.
Initiator Solution	IS	112.5 mg Michler’s Ketone dissolved in 10 mL diglyme.
Functional Monomer and Template Solution	FMTS	1.6587 mol dm^−3^ of functional monomer and 0.1668 mol dm^−3^ of template molecule in 5 mL of diglyme.

**Table 2:** Final spin-coating solution.

Solution	NPS (µL)	IS (µL)	FMTS (µL)	Diglyme (µL)
Spin-Coating Mixture	500	1000	375	125

**Table 3:** Functional monomer and template combinations.

Polymer	Template Molecule	Functional Monomer
MIP1	Diphenyl phosphate	Methacrylic acid
MIP2	Triethyl phosphate	Methacrylic acid
MIP3	Trimethyl phosphate	*N*-allylthiourea
